# Stereotactic ablative radiotherapy for treating primary head and neck cancer and locoregional recurrence: A comprehensive review of the literature

**DOI:** 10.1016/j.ctro.2024.100766

**Published:** 2024-03-29

**Authors:** Ciro Franzese, Panagiotis Balermpas

**Affiliations:** a Department of Biomedical Sciences, Humanitas University, 20090 Pieve Emanuele, Italy; b Department of Radiotherapy and Radiosurgery, IRCCS Humanitas Research Hospital, Via Manzoni 56, 20089 Milan, Italy; c Department of Radiation Oncology, University Hospital Zurich and University of Zurich, Rämistrasse 100, Zurich 8091, Switzerland

**Keywords:** Stereotactic ablative radiotherapy, Stereotactic body radiotherapy, Head and neck cancer, Squamous cell carcinoma, Local recurrence, Reirradiation, Laryngeal carcinoma, Carotid blow out, Robotic radiosurgery

## Abstract

**Introduction:**

Although stereotactic ablative radiotherapy (SABR) has advance to standard-of-care for many different indications like lung and liver malignancies, it still remains in its infancy for treating head and neck cancer. Nevertheless there is a growing body of experience and evidence, which is summarized in this review Methods A thorough search of the literature was performed and critically reviewed both for SABR as a primary treatment as well as for treating locoregionally recurrent disease in a pre-irradiated field.

**Results:**

There exist only few prospective data published so far for treating head and neck cancer with SABR. In the primary situation especially implementing SABR as a boost after definitive radiotherapy or a single-modality for locally limited, small glottic cancer appear promising. On the other hand, SABR can be a useful modality for treating local recurrence in a pre-irradiated field. However, caution is needed in the case of proximity to a pre-irradiated carotid artery or other serial organs at risk. Usually only limited gross volumes are treated with 3-6 fractions every other day and a cumulative dose of 24-44 Gy in dedicated radiosurgery platforms or modern linacs with the possibility of online image-guidance and adequate immobilsation.

**Conclusions:**

SABR is an innovative, effective and promising treatment modality for small targets, especially in near proximity to organs at risk or in a pre-irradiated region. Prospective trials are further needed for this technique to become standard-of care.

## Highlights

• The indication for stereotactic ablative radiotherapy in head and neck remains rare.
• Emerging data demonstrate a possible utility of SABR as a boost.
• SABR can be a promising modality for small, localized glottic cancer.
• There is growing evidence for SABR for re-irradiation in the head and neck.
• SABR is usually applied for small volumes, in 3–6 fractions with 24–44 Gy.

## Introduction

Head and neck cancers (HNC) represent the seventh most common tumor worldwide, with over 660.000 new cases per year globally[1], [2]. Despite notable progress in treatment modalities featuring innovative therapeutic approaches, managing primary and locoregionally recurrent disease still remains an ongoing challenge. Traditionally, HNC have been managed through surgery, external beam radiation therapy (EBRT), and systemic therapy, either alone or as a combination. In particular, EBRT is employed for radical or post-operative intent, and often involves a prolonged treatment regimen lasting six to seven weeks. While effective, these strategies frequently carry significant morbidity and can potentially compromise the patient’s quality of life (QoL), especially in cases involving elderly or frial patients, or those with multiple comorbidities.

Furthermore, the treatment choice available for patients with recurrent tumors may be limited by prior radiation therapy, which complicates the feasibility of additional EBRT due to the risk of severe toxicity to the adjacent healthy tissues. In this context, the use of stereotactic ablative radiotherapy (SABR), an advanced radiation technique, has gained attention for its capacity to administer highly ablative doses over a limited number of fractions, while precisely sparing the surrounding healthy tissue[3]. SABR has now gained acceptance for various early-stage primary tumors, and oligometastases[4], [5], [6]. This approach yields commendable rates of tumor control and mitigate the toxicity to critical OARs, making it an appealing therapeutic option for HNC patients, particulary those who are elderly, inoperable, or experiencing recurrence[7].

This indication remains in its infancy. However, during the last years, different studies have been published evaluating the safety and efficacy of SABR in different histological subtypes of HNC, even if commonly with small sample sizes, heterogeneous populations, and different radiotherapy techniques[8]. This manuscript presents a comprehensive review of the existing literature concerning applications of SABR in the management of both primary and recurrent HNC.

## 1 SABR for primary HNC

Not all HNC patients are suitable candidates for a conventional long-course-RT approach, due to factors such as age, frailty, or the presence of multiple comorbidities. In such cases, SABR may be considered. While SABR offers reduced impact on QoL and may be particularly beneficial for patients unable to tolerate more aggressive therapies, its role as a primary treatment requires careful consideration, as the majority of existing evidence is of low level and/or applies to the palliative and re-irradiation setting[9], [10], [11]. Furthermore, the use of SABR as a boost in treatment for locally advanced disease presents a novel approach, albeit with limited supporting data[12].

### Retrospective data for definitive SABR

#### Indications

In recent years, the role of SABR has been investigated in the management of primary HNC of various histologies, encompassing both a radical “stand-alone” approach and ist use as a sequential boost (Table 1)[13], [14], [15], [16], [17], [18], [19], [20], [21], [22], [23], [24], [25], [26]. The majority of these studies are retrospective, and involve a heterogeneous population with varied primary tumor sites, including the pharynx, larynx, oral cavity, sinus and salivary glands. To date, there is insufficient high-level evidence to establish this treatment as standard of care.

**Table 1:** Selected studies of SABR for primary HNC.

**Study**	**n**	**primary**	**RT intent**	**SABR dose** **(Gy)**	**N fractions**	**LC rates**	**OS rates**
**Retrospective data**							
Siddiqui, 2009[13]	10/44	mixed	radical	18–48	1–6	83.3 % 1y	50 % 2y
Kodani, 2011[14]	13/34	mixed	radical	19.5 – 42	3–8	NR	58.3 % y2
Karam, 2012[15]	13	parotid	radical - adjuvant	25–40	5–8	84 % 2y	46 % 2y
Kawaguchi, 2012[16]	14	mixed	radical	35–42	3–5	71.4 % 3y	78.6 % 3y
Amini, 2014 [17]	3	mixed	radical	25–36	5	NR	NR
Vargo, 2014[18]	12	mixed	radical	44	5	69 % 1y	64 % 1y
Gogineni, 2020[19]	66	mixed	radical	35–40	5	73 % 1y	64 % 1y
Al-Assaf, 2020 [20]	48/114	mixed	radical	35.6–53.8	4–6	85.8 % 1y	NR
Chen, 2006 [21]	64	nasopharynx	boost	12–15	4–5	93.1 % 3y	84.9 %3y
Lee, 2012[22]	26	mixed	boost	10–25	2–5	86.4 % 2y	61.5 % 2y 46.2 % 5y
**Prospective data**							
Schwartz, 2017[23]	20	larynx	radical	42.5	5	82 % 1y	100 % 1y
Sher, 2019 [24]	12/29	larynx	radical	42.5	5	100 % 2y	NR
Sanguineti, 2023 [25]	33	larynx	radical	36	3	early closure	
Vempati, 2020 [26]	34	oropharynx	boost	8–10	1–2	85.3 % 4y	85.3 % 4y

Regarding SABR for parotid gland tumors, Karam et al.[15] included patients both in the definitive (not candidates to surgery) and adjuvant setting (gross residual or adverse features). Thirteen patients with different malignant salivary gland histologies were treated with SABR, with a median dose of 33 Gy (ranging from 25 to 40 Gy). With a median follow-up of 14 months, one patient experienced local failure and four patients had distant progression. The treatment was well tolerated, with grade 1 dysphagia in six cases, and fibrosis and trismus observed in only one case.

#### Dose and prescription

In the radical setting, SABR dose ranges from 18 to 53.8 Gy, with the number of fractions varying from one to eight. The gross tumor volume (GTV) was mostly small and reported in five studies with a median value of 33.2 cc (range 22–––82). Concomitant systemic therapy for squamous cell carcinoma (SCCHN) was reported by Vargo et al.[19] in 25 % of treated patients, and by Gogineni et al.[19] in 52 % of the population. Notably, both of these studies included cohorts of elderly/ frail patients, with overall local control (LC) rates at one year ranging from 69 % to 86.4 %, and overall survival (OS) from 46 % to 61.5 %. These finding emphasize the efficacy of this modality for unfit and/or medically inoperable patients.

#### Toxicity

Regarding toxicity after radical treatment, most studies report grade 1 and grade 2 side effects, commonly including mucositis, dermatitis and dysphagia, which can be managed with supportive treatments. Moderate and severe toxicity, especially grade 4–5, appears to be quite rare when SABR is used as a single modality for previously non-irradiated patients. Siddiqui et al.[13] showed two cases (20 %) of grade 3 side effects (facial pain and cataract) in the primary treatment group. Vargo et al.[18] reported two patients (16 %) with grade 3 dysphagia and grade 3 mucositis, respectively. Moreover, the authors evaluated patient-reported QoL, and the questionnaires showed an improved or stable overall QoL in 71 % of the patients. In the study by Gogineni et al.[19], two patients (3 %) experienced grade 3 acute toxicity, including grade 3 dysphagia and grade 3 anorexia. Al-Assaf et al.[20] reported two de-novo treated patients (4 %) who developed grade 4 late toxicities in the form of osteoradionecrosis and skin ulceration. Kodani et al.[14] demonstrated significant predictive factors for OS after definitive SABR, including the absence of prior radiotherapy within the previous 24 months and smaller target volume. No other predictive factors related to OS or LC were reported in the analyzed definitive studies.

### Prospective data for definitive SABR in laryngeal carcinoma

Early stage glottic cancer is commonly managed with minimally invasive surgery or definitive RT that includes limited treatment volume without nodal irradiation compared to more advanced disease. For these reasons, glottic tumors could be potentially ideal sites for SABR. The group of Sher et al.[23], [24] enrolled 29 patients with Tis - T2 glottic cancer in a dose-escalation trial (four patients received 50 Gy in 15 fractions; 13 patients received 45 Gy in 10 fractions; 12 patients received 42.5 Gy in 5 fractions). Two actively smoking patients developed dose-limiting toxicities, with one patient (8.3 %) treated in the SABR group experiencing grade 3 laryngeal necrosis and dysphagia. The patient was treated for a T2 glottic tumor with a planning target volume of 21.3 cc. Due to the small number of events, no variables were identified as predictors of severe toxicity. Overall, the authors observed five local recurrences, none in the 5-fractions group. Sanguineti et al.[25] reported the reults of their phase II trials of SBRT for T1 glottic cancer. A total of 33 patients were treated with 36 Gy in 3 fractions with Linac-based SBRT using VMAT technique. All patients achieved excellent disease control with 100 % of LC. However, 6 patients (18.2 %) developed soft tissue necrosis (4) or cartilagenecrosis (2), thus accrual was discontinued due to concerns on late toxicity.

#### SABR as a sequential boost in nasopharyngeal carcinoma and SCCHN

Dose-escalation with conventional RT in SCCHN or nasopharyngeal cancer has historically been limited by the presence of OARs in close proximity to primary tumors, often characterized by complex shapes. Given the dose–response relationship between RT and local control, SABR has been investigated in recent years as a sequential boost in locally advanced HNC to enhance LC. However, toxicity data published so far for this approach appear contradictory, with increased severe sequelae observed in some studies. Nonetheless, this can be attributed at least partially to extreme dose-escalation and not specifically to the SABR technique.

SABR boost was eployed after conventional RT for nasopharyngeal tumors in the cohort of Chen et al.[21] which included 64 patients treated with doses of 12–15 Gy in 4–5 fractions. SABR was administered within one week after the completion of 2D-RT. The prescribed dose was administered to the periphery of the original lesion, mostly corresponding to the 85 % isodose (range 75–90 %). The 3-year LC rate was 93.1 %, regional control was 91.4 %, and OS 84.9 %, with no grade 4 acute or chronic RT-related complications. Lee et al.[22] treated 26 HNC patients (10 nasopharynx, 16 other sites) with a SABR boost; the SABR GTV ranged from 6.9 to 69.4 cc (median 28.2 cc), and the median SABR dose was 21 Gy delivered in 2–5 fractions. Cumulative BED10 from EBRT plus SABR ranged from 72.7 to 118.5 Gy (median 94.9 Gy). Complete response after treatment was observed in 21 patients (80.8 %), while severe late toxicities occured in nine patients (34.6 %), including six cases of grade 3 and one patient with grade 4 side effects. Larger GTV was significantly associated with the onset of late toxicities (p = 0.038), while SABR fractional dose was a marginally significant factor (p = 0.058). Moreover, the authors reported two deaths due to SABR-related late toxicities, one from prolonged poor oral intake due to a non-healing mucosal ulcer, and one from neurologic deteriorations arising from pontine necrosis.

A radiosurgery boost for oropharyngeal cancer was prospectively evaluated in a phase I trial conducted by Vempati et al.[26]; 34 patients with intermediate- or high-risk squamous cell carcinoma were treated with 8 Gy in 1 fraction (11), 10 Gy in 1 fraction (16), and 10 Gy in 2 fractions (7). Four patients (11.7 %) experienced tumor necrosis causing grade 3 dysphagia, of whom 3 developed grade 4 pharyngeal hemorrhage requiring surgical intervention.

## 2 SABR als re-irradiation for recurrent cases

### Indications, patient selection and oncological results

Re-irradiation (Re-RT) for locoregional recurrent SCCHN and nasopharyngeal cancer, especially in inoperable cases, has gained significant traction in recent years, encompassing Type I and Type II Re-RT according to the ESTRO/EORTC consensus definition[27]. While showing promising outcomes, this approach is linked to severe sequela[27], [28]. However, it should be kept in mind that after Re-RT the risk of progression or death is approximately four times the risk or radiation-induced severe late toxicity[29]. Throughout the 20th century, the role of Re-RT has been mainly investigational. However, modern techniques like SABR have managed to minimize dose exposure to normal tissues[30], while increasing target-dose delivery[31] and reducing toxicity[32], [33] thereby permitting Re-RT to establish a role in treating locally recurrent HNC[34]. Nevertheless, given the still compromised survival rates, the decision whether to apply Re-RT or not, must be made after careful patient selection and shared decision-making, considering the patients priorities regarding QoL. Notably, the latter has been shown to clearly increase for survivors after an initial decline within the first month following SABR[35].

Taken the above considerations into account, the American MIRI (Multi-Institutional Reirraddiation) collaborative published the results of large multi-center cohorts reporting the experience and recommendations regarding volume, dose and fractionation[36] and the optimal-patient selection[29] for the Re-RT setting: Ward et al. used recursive partitioning analysis for survival and could differentiate amongst three patients groups with clearly distinct outcomes as potential candidates for Re-RT. Of these groups, especially patients with surgically treated recurrencies and an interval of over two years to the initial irradiation course were the most suitable candidates for Re-RT, with a 2-year-OS of 61.9 %. Also, the second group with the same, long interval but unresected tumors, or shorter interval but without organ dysfunction, showed a decent survival rate of 40 %. In contrast to that, the rest of the patients (e.g. both short interval to previous irradiation and organ dysfunction) did not show any particular benefit from Re-RT [29]. Caudell et al. could demonstrate that an elective treatment was not associated with any benefit in terms of tumor control and survival and that an EQD2-dose of at least 66 Gy correlated with improved outcomes[36]. Both these properties (avoidance of any elective volumes and possibility to deliver higher dose with precision) are features provided by modern SABR, showing that it could serve as an appropriate technique for Re-RT. Matching these findings, the recent consensus-based summary of the American Radium Society (ARS) concerning appropriateness criteria for Re-RT recommends a strict selection of patients based on features such as the interval between RT-courses, disease extent and organ dysfunction [28]. Furthermore, the committee stressed the importance of tumor resection as treatment of choice for resectable cases, without reaching consensus on the value of postoperative Re-RT. In contrast to that, the appropriateness of Re-RT as treatement for unresectable disease was unambiguously accepted, with the important remark that am EQD2 dose of 60–70 Gy should be applied.

To date, no prospective comparison of distinct radiation modalities for Re-RT has been conducted. Among the existing comparative studies, one encompassing a larger patient cohort presented the data from 217 patients treated with IMRT and 197 with SABR[37]. IMRT demonstrated improved tumor control and survival for the prognostic more favorable cohort (longer time-interval between RT-series or without organ dysfunction). Nonetheless, further analyses showed no significant differences for patients treated with doses ≥ 35 Gy for smaller volumes and for patients with poor prognosis, when compared to SABR. Interestingly, severe acute toxicity ≥ 4 was significantly more prevalent in the IMRT group (5.1 % vs 0.5 %). The authors concluded that both techniques are feasible and yield superior outcome when compared to older, historical modalities. Nevertheless, the ARS recommendations already mentioned above could not yet reach a consensus about the appropriateness of SABR compared to normofractionation in this setting, although there seemed to be less ambivalence concerning the suitable stereotactic dose of 35–44 Gy [28].

Squamous cell carcinoma (SCC) patients with isolated local recurrence comprise a challenging and difficult-to-treat cohort, with extremely compromised survival, that do not seem to benefit from advancements in systemic treatment, such as the addition of cetuximab or pembrolizumab to standard chemotherapy as can be seen in the subgroup-analyses of the respective landmark trials[38], [39]. Most modern systemic approaches lead to objective response rates < 36 % and 1-year OS < 50 %. A systematic review of studies exploring the role of SABR for Re-RT demonstrated at least acceptable oncological outcomes when compared to current series of systemic treatment, with a 61.7 % clinical response rate, 2-year OS of 30 %, and even long-term survival for selected patients[40]. Yet, caution is warranted when interpreting these results, as patients selected for Re-RT have often a series of positive features compared with patients receiving systemic treatment only, the low tumor burden being the most prominent. Importantly, the majority of these studies treated small tumors with GTV mostly < 30 cc and with heterogeneous but usually larger intervals (>9 months in prospective series) between the two radiotherapy courses. In a large retrospective analysis of 132 patients, even tumor volumes of > 25 cc were already associated with compromised outcome[41]. Furthermore, one of the contemporary studies, focused solely on very small recurrencies (median PTV = 16.9 cc), demonstrated the most favorable results to date, with a median OS of 44.3 months[42]. Some series combined SABR with systemic treatment, mainly anti-EGFR agents, without observing any unexpected toxicity. An overview of the three prospective studies and the larger retrospective studies published so far can be found on Table 2[41], [42], [43], [44], [45], [46], [47].

**Table 2:** Selected studies of SABR used for re-irradiation in head and neck cancer.

**Study**	**n**	**mean dose prior RT** **(Gy)**	**dose Re-RT** **(Gy)**	**conc.** **systemic treatment**	**Grade** ≥**3 toxicities** **%**	**Median OS** **months**
***prospective data***						
*Lartigau, 2013*[43]	*56*	*NR*	*36*	*cetuximab*	*2*	*11.8*
*Yazici, 2013*[44]	*75*	*65*	*30*	*–*	*NR, 15 % CBO*	*14*
*Vargo, 2015*[45]	*50*	*70*	*44*	*cetuximab*	*6.3*	*10*
***retrospective data***						
*Vargo, 2014*[41]	*132*	*68*	*44*	*cetuximab*	*0*	*7*
*Kress, 2015*[46]	*85*	*68*	*30*	*70 %, various*	*5.9*	*17.3*
*Yamazaki, 2016*[47]	*107*	*60*	*30*	*–*	*20.6*	*14.4*
*Diao, 2021*[42]	*137*	*69*	*45*	*70 %, various*	*45*	*44.3*

Gy: Gray, conc.: concurrent, OS: overall survival, NR: not reported, CBO: carotid blow out.

### Adverse events and the risk for carotid blow-out

The overall rate of grade 3–4 adverse events in most published retrospective series does not exceed 3–9 %[33]. Nevertheless, while severe sequelae associated with stereotactic Re-RT remain rare, some feared, potentially fatal toxicities can occur, with carotid blow-out bleeding being the most prominent concern[7], [48]. Several authors reported such events occurring in more than 15 %[49] of patients irradiated with SABR for a second in-field-course. While high-level evidence remains limited, the frequency of this specific side effect seems to be higher than previously reported after using normofractionated/hyperfractionated regimens[50]. However, after these sobering events in earlier SABR reirradiation trials, both the Turkish group that published the results and Yamazaki et al. from a Japanese multicenter cohort demonstrated that simple strategies, such as every-other-day irradiation and selecting patients without carotid invasion (i.e. envelopment of < 180°) could effectively prevent most fatal events[44], [51]. Similar results were reported from the University of Pittsburgh, where, after implementing a two-fractions-per-week regimen, only 5.3 % of grade 3–4 bleeding events were observed[48]. Moreover, although there was no clear correlation of dose to the carotid and severe bleeding events, the latter occurred only in patients with Re-RT carotid D0.1 cc of 47.6 Gy or more [52]. Taken together, parameters associated with lower rates of severe bleeding events include treatment every 2nd or 3rd day, <180° circumferential tumor involvement of the artery, and no surgical intervention before or after Re-RT[53]. This was one of the main conclusions of the Hypofractionated Treatment Effects in the Clinic (HyTEC) report published in 2021, where data of 238 major vessel point doses were pooled together and modeled. This logistic model resulted to a similar Dmax in five fractions with a TD_50_ of 45.6 Gy [53].

Data about other severe sequelae are even more rare, but in general caution is warranted when applying SABR for Re-RT in close proximity to sensitive, serial organs such as nerves and cartilage. Ling et al. reported a small series of patients treated with this approach for laryngeal and hypopharyngeal recurrencies and experiencing 41.7 % late grade 3–4 toxicity compared to only 0–7 % in patients treated in other anatomical sites[54]. This risk reaches up to 11.4 % with laryngeal D5cc of 20 Gy and 25.3 % with a D5cc of 40 Gy. Nevertheless, as no reporting standards for Re-RT exist, and even less for SABR in this setting, no conclusive recommendation can be made to this regard[27], [53].

To summarize, with median 1-year OS rates of 40–55 % and acceptable overall severe toxicity rates, usually below 10 %, SABR has advanced into a reasonable treatment option for strictly selected patients with recurrence and second malignancies in a previously irradiated field in the head and neck region. Importantly, these data should be interpreted carefully, as they mostly originate from retrospective, small series of heterogeneous cohorts and with selection bias included.

**Fig. 1:**
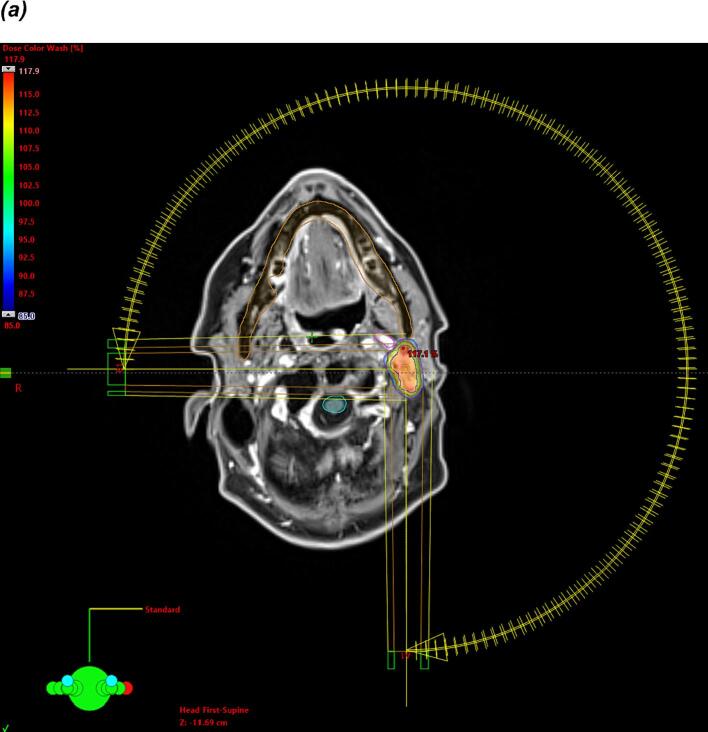

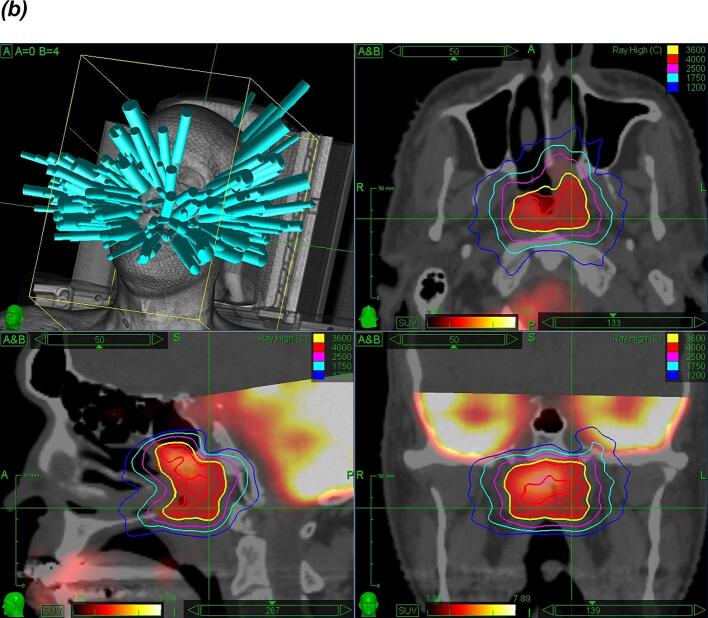
Examples of recurrent tumors treated with stereotactic re-irradiation with a prescription of 6x 6 Gy prescribed on the 85 %-isodose. (a) Excerpt from a linac-based plan of stereotactic radiotherapy with two arcs for a recurrent lymph node after registration with a planning MRI and contouring of the carotid artery (b) Excerpt from a plan of robotic radiosurgery at the Cyberknife-platform with 209 beams from 75 nodes for a recurrent nasopharyngeal cancer after registration with an FDG-PET-CT scan.

Fig. 1 illustrates two examples of recurrent HNC treated with stereotactic re-irradiation.

## 3 Ongoing trials and current trends

Although previous results described above appear promising and there are several questions to answer before SABR becomes “standard-of-care” for primary tumors (as sole modality or boost) and for re-irradiation regimens, only small prospective trials are currently open. Table 3 provides an overview of novel trials involving SABR in the treatment of head and neck cancer.

**Table 3:** Selected recruiting and planned trials on SABR for head and neck cancer.

**Study-ID** **&** **sponsor**	**Phase**	**n**	**indication**	**syst. treat.**	**primary outcome**	**status**
NCT03164460 M.D. Anderson	II rand.	100	recurrence	–	toxicity CTCAE grade 3+	recruiting
NCT05674396 M.D. Anderson	II rand.	108	palliation	–	MDASI-HN	recruiting
NCT03892720 Jonsson CCC	II	53	recurrence	–	LCR, toxicity CTCAE grade 3+	recruiting
NCT04830267 Chongqing University	II rand.	70	recurrent/ metastatic	camrelizumab	Best overall response	recruiting
NCT03546582 RTOG	I-II rand.	102	recurrence	Arm I: pembrolizumab	PFS	recruiting
NCT04576091 NCI	I	37	recurrence	pembrolizumab elimusertib	MTD of elimusertib, CTCAE	recruiting
NCT04938609 Providence H&S	II	28	neoadjuvant curative	pembrolizumab	MPR-pCR	recruiting
NCT05053737 University of Colorado	I-II	46	neoadjuvant curative	atezolizumab	I: safety (AE) II: MPR	recruiting
NCT05787639 University of California	II	29	neoadjuvant curative	evorpacept pembrolizumab	MPR-pCR	in preparation
NCT03401840 GORTEC	II	90	postoperative	–	toxicity CTCAE grade 3+	active not recruiting
NCT03114462 M.D. Anderson	I	11	definitive curative	–	toxicity CTCAE	recruiting
NCT04178174 CHUM	II rand.	106	Boost definitive curative	standard cisplatin during CRT-phase	LCR	recruiting

syst.treat.: systemic treatment; rand.: randomized; MDASI-HN: MD Anderson Symptom Inventory -Head and Neck; LCR: local control rate; PFS: progression free survival; MTD: maximum tolerated dose: MPR: major pathologic response; pCR: pathologic complete remission; CRT: chemoradiotherapy.

All of the open or recently closed trials are either phase I-II or observational trials (phase IV) with low numbers of patients and unfortunately able to provide only a limited level of evidence. Moreover, many of these trials had to be terminated due to slow patient enrollment. In a small phase IV trial, reporting results of 19 cases with various histologies, treated with heterogeneous doses of SABR no severe adverse events were observed (NCT01344356). The recruiting phase II SOAR-HN randomized trial of the M.D. Anderson Cancer center will provide more insights into the exact toxicity risks, as it compares Re-RT with SABR to standard intensity-modulated photon or proton re-treatments with primary outcome the incidence of grade 3 + events (NCT03164460). In an approach with different endpoints, the ongoing FAST phase II randomized trial of the same institution investigates the value of a 3–5 fraction SABR as palliation compared to standard palliative regimens exclusively for head and neck cancer (NCT05674396). Again, in the recurrent situation, but in non-randomized mode, the NCT03892720 trial will assess not only toxicity, but also LC rate as co-primary endpoint after treatment with HyperArc-based SABR.

A phase I trial conducted in the H. Lee Moffitt Cancer Center and Research Institute investigates SABR dose escalation for Re-RT starting with 5 times 6 Gy concomitant to low-dose cisplatin (NCT02158234). Although recruitment is completed or near completion, no results have been posted yet. A similar study from the University of Cleveland starting with 5x 7 Gy and aiming an escalation up to 5x 9 Gy seems to have been stopped due to slow accrual (NCT02388932).

A Belgian trial, where a more granular dose-escalation was planned with dose-painting on four levels from 2x 6–8 Gy up to 3x 6–12 Gy together with immunomodulating treatment with nivolumab or cyclophosphamide (NCT03402737), could also not be completed. However, the idea of exploiting the immunomodulatory effects of SABR through combinations with immunotherapy is currently being tested a.o. in a Chinese randomized phase II trial for recurrent/metasrtatic patients with at least two metastatic lesions, testing the PD-L1 inhibitor Camrelizumab with or without SABR with 27 Gy in 3 fractions to a single lesion, where also patients with locoregional lesions are included (NCT04830267). Moving away from the “abscopal” response, the RTOG-KEYSTROKE phase I-II trial prescribes SABR with or without Pembrolizumab to assess feasibility and PFS (NCT03546582). The NCT04576091 trial of the National Cancer Institute examines even the feasibility of a triple combination of SABR, pembrolizumab and the small molecule elimusertib for treating local recurrence.

Furthermore, the NIRT 2-HNC trial aims at immune-sensitisation in the curative neoadjuvant setting by applying 3x 8 Gy together with pembrolizumab in stage III-IVA primary tumors (NCT04938609). A very similar approach, but concomitantly to the anti-PD-L1 agent Atezolizumab prior to surgery has been implemented by the University of Colorado (NCT05053737). The idea of neoadjuvant, immune-sensitizing treatment, even by sparing the regional lymphatics for early tumors will be addressed also for HPV-positive oropharyngeal cancers through a combination of SABR, pembrolizumab and the fusion protein evorpacept in a phase II study (NCT05787639).

Comparable concepts applying 5–6 fractions are also tested in the postoperative setting. One of the first studies has been conducted by the University of Pittsburgh (NCT02516969), however results have been posted for only three irradiated patients. The French GORTEC group has designed a phase II trial evaluating for the first time postoperative SABR with 6 times 6 Gy for T1-T2 tumors of the oropharynx and the oral cavity with high-risk margins[55] (NCT03401840), although no results are presented yet.

In the definitive curative setting, the dose-finding HYDRA trial applies SABR for laryngeal carcinoma starting with 40 Gy in 5 fractions (NCT03114462). The use of a stereotactic boost together with de-escalated chemoradiotherapy could be another innovative approach and is currently being pursued in a randomized setting in the University of Montreal (NCT04178174).

Finally, implementing SABR for targeting distant metastases of SCCHN represents an emerging indication that has already found its way in clinical practice, especially for oligometastatic disease. Although the benefits of this strategy in this specific disease have not been conclusively proven yet, possible goals aside from prolonging survival could be a deferral of or change of systemic treatment as well as improvement in quality of life. Recently, the French GORTEC-group presented the first prospective results of a randomized trial, where SABR as first-line treatment for patients with one to three metastases was associated with reduced toxicity without compromising survival or quality of life [56].

## 4 Technical and practical recommendations

In any case, thermoplastic masks for immobilization and either dedicated platforms such as Cyberknife (Accuray, Sunnyvale, CA, USA) for robotic radiosurgery with the possibility of orthogonal x-ray, linear accelerators equipped with stereoscopic x-ray with ExacTrac (Brainlab AG, Feldkirchen, Germany), or other forms of three-dimensional, daily image guidance (IGRT) should be used.

Typically, volumes smaller than 50 cc are treated in 3–6 fractions, with prescribed doses ranging from 24 to 44 Gy[40]. Most series have employed a relatively homogenous prescription to the 80 % or higher isodose. The size of the volume clearly correlates with the incidence of adverse events and, consequently, a boost should be always applied after response to primary treatment to the shrinked tumor. However, there is not sufficient positive data to recommend single-dose radiosurgery, both due to the enhanced toxicity observed in some small trials and radiobiological considerations, such as the lack of redistribution of tumor cells. Importantly, there seems to be a clear dose–response relationship, especially for doses higher than 30 Gy compared to < 30 Gy in 5 fractions[57]. In line with that, already in the pioneering studies from the University of Pittsburgh, a dose escalation to 40–50 Gy (compared to 15–36 Gy) in five fractions was associated with improved local contral without any occurrence of grade 4–5 toxicities[58]. The planning target volume (PTV) comprises the GTV, as defined in contrast-enhanced CT and co-registered MRI, with an isotropic expansion of 2–6 mm to the PTV. Importantly, no elective target volumes should be irradiated.

For Re-RT, registration with the previous radiotherapy plan, dose summation, and calculation of biological effective dose (BED) and/or equivalent dose in 2 Gy fractions (EQD2) for both the target volume and organs at risk should be implemented in all cases. Caution is warranted especially for the carotid arteries, which should be accurately delineated and the cumulative dose after co-registration of both plans should be considered. An exclusion of patients with tumors infiltrating the carotids or enveloping more than 180° of the vessels perimeter and an implementation of every-other-day treatment schedule can mitigate the risk for dangerous bleeding incidents. Furthermore, standardizing reporting in future studies is crucial for the safe reproducibility of the results. The recommendations of the ESTRO/EORTC consensus provide a checklist of pre-requisites for planning and reporting Re-RT in general[27], and the report of the AAPM working group about SABR for this indication in HNC[57]. According to the latter, important parameters that should be reported are the following (but not limited to): exact histology, time interval between courses, prescription, coverage and dose-volume exposure of organs at risk, PTV-proximity to organs at risk, intent of SBRT, volume of target lesion, imaging used for annotation and PTV-margins, prescribed dose and dose statistics, fractionation, concurrent systemic treatments, duration of follow up, specific time points of reported outcomes, criteria for outcome assessement, exact definition of recurrence site based on treated volume.

## Conclusions

Stereotactic radiotherapy can be considered an established treatment option for HNC, particularly in the recurrent and re-irradiation context, which accounts for approximately 90 % of its use. Encouragingly, emerging evidence from the curative setting, addressing stage I-II carcinomas, combinations with surgery and/or immunotherapy, as well as treating rare histologies, could further enhance the spectrum of indications. However, the considerable heterogeneity in patient selection, techniques and reporting hampers general implementation in clinical practice. Notably, SABR should not be used for dose-escalation without enough experience for a specific clinical indication, as enhanced toxicity might be observed. Prospective registers and trials featuring standardized cohorts and reporting of treatment characteristics are necessary in order to make this modality mature for entry in guidelines.

## CRediT authorship contribution statement

**CF:** Conceptualization, Writing – original draft, Writing – review & editing. **PB:** Conceptualization, Writing – original draft, Writing – review & editing.

## Declaration of competing interest

The authors declare that they have no known competing financial interests or personal relationships that could have appeared to influence the work reported in this paper.
